# Supplementary material for: A Likelihood Approach for Real-Time Calibration of Stochastic Compartmental Epidemic Models
Source: PLoS Comput Biol. 2017 Jan 17;13(1):e1005257. doi: 10.1371/journal.pcbi.1005257 (PMC5240920; doi:10.1371/journal.pcbi.1005257)
Supplement: S1 File — (TAR.GZ) [file pcbi.1005257.s014.tar.gz › HSPH_Online-SI-Revision/IPoi09/n10-severe/n10-severe_BENCH2_table-8.pdf]

1	newly 3 weeks spec	291.	561.821	{235., 822.}	107.18	0.33415	newly 3 weeks cumul	664.	1402.04	{489., 2281.}	127.205	0.365672	newly 5 weeks spec	473.	674.25	{444., 857.}	52.3633	0.196614	newly 5 weeks cumul	1512.	2720.04	{1286., 3695.}	91.6549	0.296762	newly 59 weeks cumul 6569. 6984.21 {4795., 8386.} 15.0295 0.0644889
2	newly 3 weeks spec	216.	379.769	{91., 569.}	93.8746	0.331125	newly 3 weeks cumul	425.	993.231	{153., 1575.}	151.421	0.43012	newly 5 weeks spec	426.	342.192	{113., 542.}	31.2658	0.226756	newly 5 weeks cumul	1146.	1711.58	{681., 2345.}	66.2941	0.270646	newly 61 weeks cumul 6678. 3161.12 {2483., 4183.} 52.6637 0.335707
3	newly 3 weeks spec	232.	528.281	{237., 954.}	135.924	0.350042	newly 3 weeks cumul	460.	1283.25	{447., 2160.}	188.315	0.427794	newly 5 weeks spec	491.	612.25	{141., 956.}	58.7576	0.256302	newly 5 weeks cumul	1296.	2503.97	{1509., 4062.}	99.0717	0.28993	newly 63 weeks cumul 5504. 4552.72 {2851., 7882.} 32.1885 0.170105
4	newly 3 weeks spec	222.	141.811	{33., 291.}	50.5354	0.443093	newly 3 weeks cumul	397.	328.792	{91., 664.}	52.7494	0.402942	newly 5 weeks spec	435.	242.887	{54., 441.}	45.63	0.466072	newly 5 weeks cumul	1174.	761.075	{200., 1462.}	47.0139	0.433016	newly 58 weeks cumul 6313. 7322.06 {6392., 8079.} 20.0282 0.161706
5	newly 3 weeks spec	228.	254.12	{126., 438.}	38.4035	0.268672	newly 3 weeks cumul	503.	778.16	{460., 1110.}	64.1511	0.332942	newly 5 weeks spec	460.	214.96	{86., 366.}	53.2696	0.489492	newly 5 weeks cumul	1330.	1231.64	{786., 1679.}	27.1008	0.271492	newly 58 weeks cumul 6047. 2166.04 {1093., 3947.} 64.1905 0.650993
6	newly 3 weeks spec	208.	107.706	{25., 231.}	58.3239	0.488611	newly 3 weeks cumul	401.	237.765	{53., 529.}	55.611	0.436938	newly 5 weeks spec	328.	194.529	{51., 413.}	51.7994	0.444706	newly 5 weeks cumul	983.	581.314	{153., 1327.}	53.4897	0.428636	newly 59 weeks cumul 5568. 7620.06 {7033., 8370.} 40.7726 0.201
7	newly 3 weeks spec	360.	600.133	{148., 825.}	83.4722	0.280241	newly 3 weeks cumul	893.	1505.37	{311., 2297.}	89.944	0.299048	newly 5 weeks spec	397.	693.35	{383., 857.}	78.9631	0.245854	newly 5 weeks cumul	1675.	2861.22	{944., 3847.}	83.599	0.27005	newly 58 weeks cumul 4740. 7040.67 {5761., 8037.} 48.7968 0.168429
8	newly 3 weeks spec	247.	316.359	{40., 684.}	82.5595	0.379217	newly 3 weeks cumul	499.	629.266	{77., 1336.}	83.1507	0.383081	newly 5 weeks spec	477.	598.344	{111., 973.}	62.7096	0.279818	newly 5 weeks cumul	1346.	1682.27	{228., 3232.}	72.1896	0.327023	newly 62 weeks cumul 5812. 8502.81 {7489., 9221.} 46.2975 0.1637
9	newly 3 weeks spec	71.	472.106	{34., 967.}	585.256	0.919475	newly 3 weeks cumul	127.	786.128	{45., 1689.}	542.084	0.910034	newly 5 weeks spec	311.	1075.	{179., 1701.}	266.087	0.791249	newly 5 weeks cumul	564.	2670.32	{369., 4392.}	394.187	0.916058	newly 66 weeks cumul 6105. 6886.94 {4432., 9351.} 37.1607 0.515495
10	newly 3 weeks spec	214.	753.403	{97., 1574.}	275.678	0.676366	newly 3 weeks cumul	335.	1248.44	{139., 2798.}	297.646	0.694134	newly 5 weeks spec	555.	1457.92	{548., 1952.}	174.615	0.583157	newly 5 weeks cumul	1279.	3926.37	{1079., 6100.}	220.753	0.656852	newly 66 weeks cumul 6231. 7836.15 {5563., 9262.} 38.1695 0.355934
11	newly 3 weeks spec	107.	613.083	{53., 1047.}	494.86	0.928863	newly 3 weeks cumul	156.	1728.17	{112., 2794.}	1026.71	1.11775	newly 5 weeks spec	375.	351.042	{146., 637.}	42.8778	0.447569	newly 5 weeks cumul	750.	2568.67	{444., 3784.}	262.733	0.767267	newly 65 weeks cumul 5214. 3415.63 {2112., 4381.} 34.4913 0.479634
12	newly 3 weeks spec	89.	239.299	{17., 517.}	199.866	0.532281	newly 3 weeks cumul	220.	428.015	{33., 888.}	136.75	0.493063	newly 5 weeks spec	266.	646.433	{108., 1178.}	166.317	0.514901	newly 5 weeks cumul	648.	1494.31	{183., 3070.}	158.617	0.517487	newly 65 weeks cumul 5759. 8144. {6158., 9301.} 49.5844 0.307246
13	newly 3 weeks spec	161.	116.935	{12., 282.}	63.0735	0.56848	newly 3 weeks cumul	272.	249.903	{43., 578.}	68.8093	0.477912	newly 5 weeks spec	444.	233.71	{38., 545.}	57.9047	0.59464	newly 5 weeks cumul	1004.	653.694	{92., 1525.}	60.0774	0.545513	newly 62 weeks cumul 6792. 7566.15 {6725., 8665.} 21.3484 0.26159
14	newly 3 weeks spec	177.	226.05	{29., 453.}	91.0452	0.486954	newly 3 weeks cumul	299.	423.95	{46., 918.}	101.99	0.453068	newly 5 weeks spec	540.	551.917	{115., 1021.}	56.6698	0.400054	newly 5 weeks cumul	1231.	1353.37	{229., 2618.}	69.6209	0.409226	newly 64 weeks cumul 5655. 7727.62 {5333., 9268.} 45.8028 0.261075
15	newly 3 weeks spec	125.	1080.83	{0., 3281.}	862.312	1.98194	newly 3 weeks cumul	202.	1612.27	{0., 5478.}	795.975	2.01584	newly 5 weeks spec	451.	786.32	{0., 2164.}	170.909	1.90529	newly 5 weeks cumul	842.	3593.45	{0., 8413.}	421.776	2.21751	newly 68 weeks cumul 6627. 4672.38 {0., 9249.} 61.5226 2.2089
16	newly 3 weeks spec	329.	602.912	{15., 984.}	106.589	0.608458	newly 3 weeks cumul	674.	1637.88	{63., 2595.}	166.844	0.683355	newly 5 weeks spec	678.	464.618	{14., 897.}	50.0824	0.630892	newly 5 weeks cumul	1835.	2666.53	{93., 4051.}	68.8251	0.54123	newly 64 weeks cumul 6828. 3802.82 {1931., 6150.} 44.6173 0.591401
17	newly 3 weeks spec	187.	638.109	{143., 1129.}	253.	0.522675	newly 3 weeks cumul	387.	1323.11	{248., 2420.}	256.359	0.518951	newly 5 weeks spec	326.	939.618	{464., 1311.}	194.239	0.453306	newly 5 weeks cumul	968.	3088.93	{1187., 4866.}	225.573	0.493509	newly 64 weeks cumul 6543. 7517.42 {4606., 9145.} 28.3698 0.117989
18	newly 3 weeks spec	201.	483.359	{65., 859.}	168.848	0.476166	newly 3 weeks cumul	460.	1078.87	{160., 2023.}	166.477	0.475672	newly 5 weeks spec	415.	665.872	{155., 1088.}	86.9941	0.325166	newly 5 weeks cumul	1164.	2344.18	{399., 3945.}	127.339	0.408936	newly 62 weeks cumul 6707. 6189.33 {4357., 8849.} 23.7008 0.115743
19	newly 3 weeks spec	266.	347.591	{224., 533.}	40.2768	0.141778	newly 3 weeks cumul	588.	1039.18	{576., 1426.}	84.3228	0.256653	newly 5 weeks spec	499.	307.136	{131., 495.}	38.8504	0.256631	newly 5 weeks cumul	1512.	1685.41	{1086., 2235.}	26.909	0.109024	newly 58 weeks cumul 6578. 3000.73 {1715., 4331.} 54.3824 0.371217
20	newly 3 weeks spec	163.	259.4	{146., 353.}	64.	0.202669	newly 3 weeks cumul	399.	818.8	{576., 1102.}	105.213	0.301561	newly 5 weeks spec	300.	198.8	{86., 330.}	41.1467	0.27059	newly 5 weeks cumul	931.	1259.48	{786., 1658.}	41.2718	0.144506	newly 58 weeks cumul 5881. 1875.88 {972., 2809.} 68.1027 0.526697
21	newly 3 weeks spec	178.	901.91	{237., 1531.}	411.84	0.648354	newly 3 weeks cumul	310.	1408.85	{336., 2445.}	360.843	0.59845	newly 5 weeks spec	488.	1701.73	{1149., 2006.}	248.715	0.531269	newly 5 weeks cumul	1116.	4596.66	{2392., 5999.}	311.887	0.585488	newly 66 weeks cumul 6367. 8666.46 {7862., 9312.} 37.4843 0.137858
22	newly 3 weeks spec	136.	705.059	{113., 1085.}	433.088	0.836933	newly 3 weeks cumul	315.	1693.97	{187., 2804.}	454.911	0.79982	newly 5 weeks spec	337.	680.088	{279., 1094.}	115.631	0.496253	newly 5 weeks cumul	870.	3116.24	{960., 4240.}	270.71	0.691556	newly 64 weeks cumul 5004. 4794.85 {3612., 5859.} 17.7793 0.267089
23	newly 3 weeks spec	247.	580.766	{83., 1050.}	155.423	0.420738	newly 3 weeks cumul	543.	1326.81	{191., 2470.}	167.301	0.435837	newly 5 weeks spec	429.	756.191	{299., 1082.}	89.9965	0.283816	newly 5 weeks cumul	1291.	2789.28	{653., 4470.}	132.329	0.374805	newly 63 weeks cumul 5813. 6690.17 {4505., 9123.} 29.396 0.116294
24	newly 3 weeks spec	98.	193.467	{0., 388.}	149.728	0.70902	newly 3 weeks cumul	155.	426.3	{9., 871.}	217.097	0.617934	newly 5 weeks spec	358.	288.967	{0., 558.}	55.8939	0.749453	newly 5 weeks cumul	700.	959.6	{9., 1991.}	93.419	0.574967	newly 64 weeks cumul 6876. 3705.8 {9., 7364.} 49.872 0.648747
25	newly 3 weeks spec	16.	774.246	{0., 2077.}	4763.83	1.62708	newly 3 weeks cumul	53.	1143.61	{11., 3045.}	2081.57	1.28912	newly 5 weeks spec	207.	1483.08	{0., 2250.}	639.416	1.1535	newly 5 weeks cumul	318.	3931.34	{11., 7377.}	1159.33	1.27524	newly 67 weeks cumul 5792. 7453.23 {11., 9333.} 53.635 0.612386
26	newly 3 weeks spec	172.	430.412	{46., 825.}	174.145	0.478193	newly 3 weeks cumul	282.	667.088	{66., 1297.}	162.756	0.45831	newly 5 weeks spec	397.	1271.18	{350., 1773.}	230.064	0.552562	newly 5 weeks cumul	956.	2802.96	{528., 4621.}	207.588	0.5283	newly 66 weeks cumul 5226. 8602.6 {7502., 9357.} 67.5528 0.286886
27	newly 3 weeks spec	68.	18.0548	{0., 45.}	82.5141	1.35917	newly 3 weeks cumul	149.	30.7397	{0., 83.}	82.5871	1.32178	newly 5 weeks spec	292.	78.5068	{0., 182.}	84.073	1.55548	newly 5 weeks cumul	586.	147.74	{0., 340.}	83.737	1.46486	newly 67 weeks cumul 6353. 5925.89 {0., 9259.} 48.9884 1.1877
28	newly 3 weeks spec	54.	84.5833	{0., 203.}	128.858	0.652768	newly 3 weeks cumul	104.	142.767	{4., 352.}	115.256	0.619742	newly 5 weeks spec	285.	292.05	{0., 821.}	89.6082	0.764309	newly 5 weeks cumul	567.	598.917	{4., 1622.}	93.0364	0.719096	newly 66 weeks cumul 5472. 7554.28 {4., 9306.} 59.3351 0.577692
29	newly 3 weeks spec	78.	10.74	{0., 24.}	94.9231	2.03684	newly 3 weeks cumul	105.	17.16	{0., 36.}	93.2381	1.80149	newly 5 weeks spec	382.	59.13	{0., 120.}	93.233	2.48121	newly 5 weeks cumul	647.	101.57	{0., 204.}	93.3308	2.25806	newly 68 weeks cumul 5977. 3412.51 {0., 9026.} 73.0284 2.37109
30	newly 3 weeks spec	42.	13.44	{0., 24.}	99.4762	1.75724	newly 3 weeks cumul	59.	21.26	{0., 40.}	97.0508	1.52615	newly 5 weeks spec	274.	86.86	{0., 172.}	102.197	2.26092	newly 5 weeks cumul	436.	148.02	{0., 260.}	102.216	2.05771	newly 68 weeks cumul 6478. 3815.73 {0., 8989.} 67.0045 2.32646
31	newly 3 weeks spec	233.	836.581	{235., 1327.}	271.092	0.640819	newly 3 weeks cumul	480.	2016.39	{1012., 3034.}	332.07	0.716855	newly 5 weeks spec	432.	632.194	{170., 1140.}	79.6147	0.384803	newly 5 weeks cumul	1241.	3435.26	{2128., 4718.}	186.244	0.561292	newly 65 weeks cumul 5273. 4546. {3164., 5891.} 29.2733 0.275922
32	newly 3 weeks spec	278.	434.259	{201., 614.}	68.0922	0.224808	newly 3 weeks cumul	694.	1440.96	{876., 1873.}	108.56	0.302495	newly 5 weeks spec	361.	303.481	{106., 624.}	43.9417	0.251016	newly 5 weeks cumul	1404.	2121.3	{1170., 2790.}	58.2964	0.195157	newly 58 weeks cumul 4849. 2924.48 {1310., 4672.} 39.689 0.257225
33	newly 3 weeks spec	255.	446.667	{186., 721.}	88.3951	0.270026	newly 3 weeks cumul	589.	1055.83	{349., 1897.}	94.6205	0.277405	newly 5 weeks spec	409.	612.278	{403., 768.}	57.4708	0.201998	newly 5 weeks cumul	1337.	2205.87	{1008., 3416.}	76.2251	0.242869	newly 58 weeks cumul 5959. 7862.87 {7149., 8529.} 31.9495 0.119127
34	newly 3 weeks spec	330.	562.3	{361., 749.}	76.1667	0.258836	newly 3 weeks cumul	818.	1626.7	{786., 2186.}	105.513	0.320935	newly 5 weeks spec	455.	512.325	{245., 754.}	36.8736	0.163019	newly 5 weeks cumul	1672.	2693.4	{1988., 3464.}	65.9629	0.230219	newly 58 weeks cumul 6027. 4764.33 {2940., 6874.} 27.3888 0.147665
35	newly 3 weeks spec	124.	404.17	{13., 861.}	265.627	0.724237	newly 3 weeks cumul	254.	753.604	{36., 1567.}	238.234	0.677591	newly 5 weeks spec	365.	798.66	{47., 1348.}	151.512	0.618457	newly 5 weeks cumul	843.	2165.77	{96., 4154.}	191.725	0.657306	newly 65 weeks cumul 5774. 7495.53 {5385., 9204.} 44.3069 0.377819
36	newly 3 weeks spec	206.	465.425	{184., 713.}	131.323	0.342538	newly 3 weeks cumul	457.	1178.45	{363., 1878.}	165.219	0.39816	newly 5 weeks spec	363.	538.3	{247., 864.}	62.8375	0.210708	newly 5 weeks cumul	1101.	2242.9	{1134., 3373.}	107.847	0.300069	newly 61 weeks cumul 6168. 4902.98 {2817., 7752.} 32.8846 0.181352
37	newly 3 weeks spec	223.	259.862	{40., 568.}	72.0386	0.355195	newly 3 weeks cumul	420.	463.585	{82., 1002.}	70.7509	0.357744	newly 5 weeks spec	351.	667.831	{153., 1170.}	113.899	0.361771	newly 5 weeks cumul	1055.	1574.43	{286., 3000.}	84.2129	0.324512	newly 64 weeks cumul 5466. 8510.45 {7629., 9208.} 56.7443 0.194007
38	newly 3 weeks spec	171.	495.516	{88., 855.}	210.262	0.464742	newly 3 weeks cumul	342.	1114.13	{135., 1908.}	243.162	0.499859	newly 5 weeks spec	402.	582.323	{202., 918.}	65.126	0.224558	newly 5 weeks cumul	991.	2276.97	{651., 4014.}	144.211	0.370586	newly 64 weeks cumul 6742. 4235.55 {2955., 5401.} 38.457 0.22575
39	newly 3 weeks spec	270.	526.257	{155., 810.}	112.519	0.332605	newly 3 weeks cumul	603.	1261.49	{274., 1957.}	129.206	0.3675	newly 5 weeks spec	373.	591.857	{360., 838.}	65.6453	0.215142	newly 5 weeks cumul	1298.	2425.46	{900., 3921.}	100.178	0.301268	newly 60 weeks cumul 6183. 5671.77 {4000., 7503.} 22.8253 0.108359
40	newly 3 weeks spec	213.	283.593	{141., 445.}	56.0598	0.219049	newly 3 weeks cumul	503.	859.889	{286., 1321.}	90.0376	0.293762	newly 5 weeks spec	419.	259.407	{78., 425.}	41.112	0.282358	newly 5 weeks cumul	1236.	1380.56	{855., 1983.}	37.8671	0.169007	newly 60 weeks cumul 6409. 2624.56 {1281., 3623.} 59.0489 0.415255
41	newly 3 weeks spec	69.	69.84	{0., 74.}	165.362	1.85106	newly 3 weeks cumul	109.	94.95	{0., 101.}	148.45	1.73614	newly 5 weeks spec	305.	215.27	{0., 433.}	132.37	2.25625	newly 5 weeks cumul	561.	461.16	{0., 673.}	143.34	2.12105	newly 68 weeks cumul 6138. 3924.77 {0., 9169.} 68.238 2.21697
42	newly 3 weeks spec	203.	46.2051	{4., 111.}	77.2389	0.947542	newly 3 weeks cumul	358.	112.179	{7., 266.}	72.7904	0.811962	newly 5 weeks spec	454.	73.	{3., 161.}	83.9207	1.08017	newly 5 weeks cumul	1061.	243.	{13., 583.}	77.0971	0.930019	newly 59 weeks cumul 6484. 5960.26 {3604., 7286.} 17.4829 0.250367
43	newly 3 weeks spec	127.	50.5075	{0., 208.}	83.1473	1.29905	newly 3 weeks cumul	210.	81.1343	{0., 308.}	79.8294	1.16266	newly 5 weeks spec	451.	225.851	{0., 832.}	87.325	1.38918	newly 5 weeks cumul	888.	422.896	{0., 1610.}	84.7956	1.31331	newly 67 weeks cumul 6305. 6240.73 {0., 9131.} 46.9682 0.992924
44	newly 3 weeks spec	233.	321.872	{71., 679.}	81.479	0.37861	newly 3 weeks cumul	482.	749.538	{147., 1724.}	100.277	0.364426	newly 5 weeks spec	403.	485.051	{188., 847.}	51.1421	0.285214	newly 5 weeks cumul	1184.	1638.36	{452., 2949.}	73.9692	0.308747	newly 61 weeks cumul 5552. 6433.41 {3800., 8307.} 33.5084 0.187584
45	newly 3 weeks spec	215.	243.7	{34., 592.}	76.4452	0.407868	newly 3 weeks cumul	394.	435.657	{65., 1089.}	76.628	0.394754	newly 5 weeks spec	444.	592.5	{123., 1099.}	69.6107	0.331762	newly 5 weeks cumul	1183.	1428.51	{272., 3037.}	69.9698	0.340608	newly 64 weeks cumul 6681. 8456.19 {7153., 9333.} 31.8496 0.162013
46	newly 3 weeks spec	191.	427.032	{59., 659.}	153.64	0.527079	newly 3 weeks cumul	452.	1102.26	{170., 1888.}	173.951	0.565613	newly 5 weeks spec	337.	450.839	{132., 704.}	66.7656	0.357708	newly 5 weeks cumul	1052.	2022.13	{386., 2858.}	119.073	0.487899	newly 63 weeks cumul 5089. 3743.06 {2387., 5011.} 27.2885 0.282795
47	newly 3 weeks spec	210.	455.357	{165., 689.}	134.184	0.563939	newly 3 weeks cumul	472.	1267.43	{283., 1774.}	188.529	0.6779	newly 5 weeks spec	303.	337.643	{91., 881.}	69.5662	0.504374	newly 5 weeks cumul	1066.	2017.	{633., 2885.}	109.3	0.582071	newly 64 weeks cumul 5261. 2827.43 {1615., 4709.} 46.2568 0.574651
48	newly 3 weeks spec	153.	137.97	{26., 312.}	62.3292	0.375923	newly 3 weeks cumul	281.	282.136	{47., 696.}	66.2299	0.361668	newly 5 weeks spec	442.	289.879	{67., 612.}	49.7463	0.370041	newly 5 weeks cumul	972.	781.076	{165., 1763.}	55.0864	0.357593	newly 61 weeks cumul 6845. 8344.5 {7547., 8868.} 21.9065 0.0851664
49	newly 3 weeks spec	191.	446.759	{30., 695.}	161.022	0.543656	newly 3 weeks cumul	421.	1148.55	{54., 1897.}	202.515	0.58036	newly 5 weeks spec	403.	487.621	{155., 806.}	50.1925	0.312855	newly 5 weeks cumul	1126.	2135.45	{274., 3089.}	114.657	0.45092	newly 63 weeks cumul 5314. 3927.52 {3059., 5111.} 27.5914 0.253218
50	newly 3 weeks spec	165.	333.56	{0., 1555.}	240.982	2.0026	newly 3 weeks cumul	248.	441.57	{0., 1971.}	217.069	1.94576	newly 5 weeks spec	521.	544.6	{0., 1830.}	134.699	2.19742	newly 5 weeks cumul	1082.	1525.7	{0., 6489.}	172.414	2.22769	newly 68 weeks cumul 6795. 3955.67 {0., 9123.} 63.9845 2.33173
